# Bis(μ-2-phenyl­acetato-κ^2^ *O*:*O*)bis­[(2,2′-bipyridyl-κ^2^ *N*,*N*′)(2-phenyl­acetato-κ*O*)copper(II)] dihydrate

**DOI:** 10.1107/S1600536811035483

**Published:** 2011-09-14

**Authors:** Wei Xu, Ling Jin, Bin-Bin Liu

**Affiliations:** a Center of Applied Solid State Chemistry Research, Ningbo University, Ningbo, Zhejiang 315211, People’s Republic of China

## Abstract

The mol­ecule of the binuclear title complex, [Cu_2_(C_8_H_7_O_2_)_4_(C_10_H_8_N_2_)_2_]·2H_2_O, is located on an inversion centre. The Cu atoms are bridged by two O atoms of the monodentate phenyl­acetate groups [Cu—O = 1.9808 (14) and 2.3668 (14) Å]. The longer of the two bridging Cu—O bonds takes the apical position of the distorted square-pyramidal environment of the Cu atom, which is completed by two N atoms of the chelate 2,2′-bipyridine ligand [Cu—N = 2.0107 (17) and 2.0234 (16) Å]. The mol­ecules are assembled into stacks along [100] through π–π inter­actions with inter­planar distances of 3.630 (4) and 3.407 (3) Å; the resulting stacks are further connected into a three-dimensional supra­molecular architecture by O—H⋯O and C—H⋯O hydrogen-bonding inter­actions.

## Related literature

For applications of inorganic–organic hybrid materials, see: Pan *et al.* (2003 ▶); Shibasaki & Yoshikawa (2002 ▶). For related structures, see: Addison & Rao (1984 ▶); Antolini *et al.* (1985 ▶); Zhang *et al.* (2006 ▶).
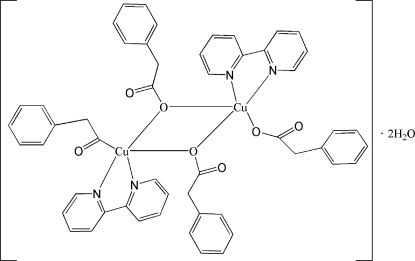


## Experimental

### 

#### Crystal data

[Cu_2_(C_8_H_7_O_2_)_4_(C_10_H_8_N_2_)_2_]·2H_2_O
*M* *_r_* = 1016.02
Monoclinic, 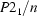

*a* = 10.213 (2) Å
*b* = 16.058 (3) Å
*c* = 14.633 (3) Å
β = 100.75 (3)°
*V* = 2357.7 (8) Å^3^
*Z* = 2
Mo *K*α radiation
μ = 0.97 mm^−1^
*T* = 295 K
0.17 × 0.14 × 0.11 mm

#### Data collection

Rigaku R-AXIS RAPID diffractometer
Absorption correction: multi-scan (*ABSCOR*; Higashi, 1995 ▶) *T*_min_ = 0.678, *T*_max_ = 0.784
22348 measured reflections
5356 independent reflections
4268 reflections with *I* > 2σ(*I*)
*R*_int_ = 0.033

#### Refinement

*R*[*F* ^2^ > 2σ(*F* ^2^)] = 0.031
*wR*(*F* ^2^) = 0.090
*S* = 1.10
5356 reflections
307 parameters
H-atom parameters constrained
Δρ_max_ = 0.29 e Å^−3^
Δρ_min_ = −0.56 e Å^−3^

### 

Data collection: *RAPID-AUTO* (Rigaku, 1998 ▶); cell refinement: *RAPID-AUTO*; data reduction: *CrystalStructure* (Rigaku/MSC, 2004 ▶); program(s) used to solve structure: *SHELXS97* (Sheldrick, 2008 ▶); program(s) used to refine structure: *SHELXL97* (Sheldrick, 2008 ▶); molecular graphics: *ORTEPII* (Johnson, 1976 ▶); software used to prepare material for publication: *SHELXL97*.

## Supplementary Material

Crystal structure: contains datablock(s) global, I. DOI: 10.1107/S1600536811035483/ya2144sup1.cif

Structure factors: contains datablock(s) I. DOI: 10.1107/S1600536811035483/ya2144Isup2.hkl

Additional supplementary materials:  crystallographic information; 3D view; checkCIF report

## Figures and Tables

**Table 1:** Hydrogen-bond geometry (Å, °)

*D*—H⋯*A*	*D*—H	H⋯*A*	*D*⋯*A*	*D*—H⋯*A*
O5—H51⋯O4	0.85	2.05	2.781 (3)	143
O5—H52⋯O2^i^	0.86	2.08	2.931 (3)	174
C20—H20*A*⋯O4^ii^	0.93	2.38	3.245 (3)	156
C24—H24*A*⋯O2^iii^	0.93	2.48	3.172 (3)	131
C25—H25*A*⋯O5^iv^	0.93	2.50	3.201 (3)	132

Symmetry codes: (i) 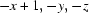; (ii) 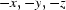; (iii) 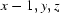; (iv) 
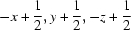
.

**Fig. 1.**
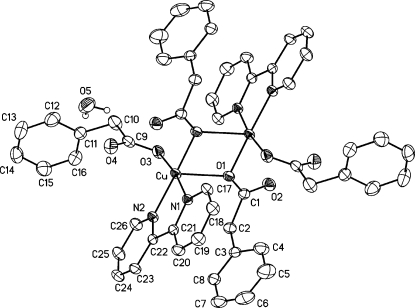
ORTEP view of the title compound. The displacement ellipsoids are drawn at 45% probability level; hydrogen atoms bonded to carbon were omitted.

**Fig. 2.**
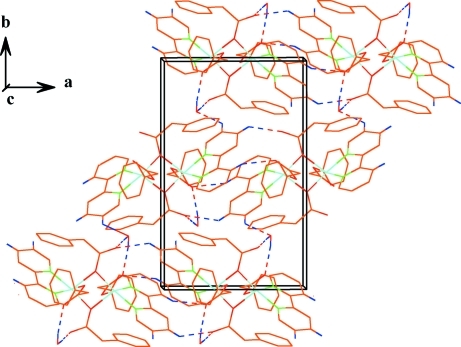
Crystal packing of the title complex viewed down the c axis. Hydrogen bonds are shown as dashed lines.

## supplementary crystallographic information

### Comment

The use of metal centers for organizing of molecular building blocks into inorganic–organic hybrid materials have been studied for potential applications in catalysis, gas storage, and in molecular–based magnetic materials (Pan *et al.*, 2003; Shibasaki & Yoshikawa, 2002). As part of our investigations of self-assemblies of Cu^2+^ ions and bipy with phenylacetic acid, we prepared the title complex, [Cu_2_(C_8_H_7_O_2_)_4_(C_10_H_8_N_2_)_2_].2H_2_O.

The molecule of the complex occupies a special position in the inversion centre (Fig. 1). The square pyramidal coordination environment of the Cu atom is formed by the N atoms of 2,2'-bipyridine ligands (Cu—N1 2.0108 (17) Å and Cu—N2 2.0234 (16) Å), the O atom of terminal phenylacetato ligand (Cu—O3 1.9557 (16) Å), and two O atoms of bridging phenylacetato groups, the O1^i^ atom [symmetry code (i): 1 - *x*, -*y*, -*z*] takes one of the equatorial positions, whereas the O1 atom occupies the apical site. As one would expect (Antolini *et al.*, 1985; Zhang *et al.*, 2006), the apical bond Cu—O1 2.3669 (14) Å is substantially longer than the equatorial Cu—O1^i^ distance of 1.9807 (14) Å. The Cu atom is displaced by 0.078 (1) Å towards the apical vertex from the mean plane of the equatorial ligands [τ = 0.04 according to Addison & Rao (1984)].

The molecules are assembled into stacks along [100] through π···π stacking interactions with the mean interplanar distance of 3.407 (3) Å between adjacent bipy ligands and 3.630 (4) Å between bipy ligands and phenylacetato groups, and the stacks are further stabilized by the weak C—H···O hydrogen bonding interactions from the phenyl CH groups to the uncoordinating carboxylate O2 and O4 atoms (Table 1), as well O—H···O bonds involving water molecule (Fig. 2). As a result, three-dimensional network is formed.

### Experimental

Phenylacetic acid(0.2726 g, 2.000 mmol) was completely dissolved in a mixture of 10 ml of ethanol, 10 ml of water, and bipy (0.1561 g, 1.000 mmol). 0.2602 g (1.084 mmol) of Cu(NO_3_)_2_.3H_2_O were then added, and after dropwise addition of 2.0 ml (1*M*) NaOH to the resulting solution under continuous stirring for 1 h, the blue suspension was produced. The suspension was filtered and the filtrate (pH = 6.51) was allowed to stand at room temperature for several weeks; the precipitation of blue block crystals was observed.

### Refinement

H atoms bonded to C atoms were placed in geometrically calculated positions (C—H 0.93 Å and 0.97 Å for aromatic and methylene H atoms respectively) and were included in the refinement in a riding model approximation, with *U*_iso_(H) = 1.2 *U*_eq_(C). H atoms attached to O atoms were found in a difference Fourier synthesis and were also included in the riding model approximation, with the O—H distances fixed as initially found and with *U*_iso_(H) values set at 1.5 *U*eq(O).

### Crystal data

**Table:**

[Cu_2_(C_8_H_7_O_2_)_4_(C_10_H_8_N_2_)_2_]·2H_2_O	*F*(000) = 1052
*M**_r_* = 1016.02	*D*_x_ = 1.431 Mg m^−^^3^
Monoclinic, *P*2_1_/*n*	Mo *K*α radiation, λ = 0.71073 Å
Hall symbol: -P 2yn	Cell parameters from 22348 reflections
*a* = 10.213 (2) Å	θ = 3.1–27.4°
*b* = 16.058 (3) Å	µ = 0.97 mm^−^^1^
*c* = 14.633 (3) Å	*T* = 295 K
β = 100.75 (3)°	Block, blue
*V* = 2357.7 (8) Å^3^	0.17 × 0.14 × 0.11 mm
*Z* = 2	

### Data collection

**Table:**

Rigaku R-AXIS RAPID diffractometer	5356 independent reflections
Radiation source: fine-focus sealed tube	4268 reflections with *I* > 2σ(*I*)
graphite	*R*_int_ = 0.033
Detector resolution: 0 pixels mm^-1^	θ_max_ = 27.4°, θ_min_ = 3.1°
ω scans	*h* = −13→13
Absorption correction: multi-scan (*ABSCOR*; Higashi, 1995)	*k* = −20→20
*T*_min_ = 0.678, *T*_max_ = 0.784	*l* = −18→18
22348 measured reflections	

### Refinement

**Table:**

Refinement on *F*^2^	Primary atom site location: structure-invariant direct methods
Least-squares matrix: full	Secondary atom site location: difference Fourier map
*R*[*F*^2^ > 2σ(*F*^2^)] = 0.031	Hydrogen site location: inferred from neighbouring sites
*wR*(*F*^2^) = 0.090	H-atom parameters constrained
*S* = 1.10	*w* = 1/[σ^2^(*F*_o_^2^) + (0.0357*P*)^2^ + 1.1037*P*] where *P* = (*F*_o_^2^ + 2*F*_c_^2^)/3
5356 reflections	(Δ/σ)_max_ < 0.001
307 parameters	Δρ_max_ = 0.29 e Å^−^^3^
0 restraints	Δρ_min_ = −0.56 e Å^−^^3^

### Special details

**Table:**

Geometry. All e.s.d.'s (except the e.s.d. in the dihedral angle between two l.s. planes) are estimated using the full covariance matrix. The cell e.s.d.'s are taken into account individually in the estimation of e.s.d.'s in distances, angles and torsion angles; correlations between e.s.d.'s in cell parameters are only used when they are defined by crystal symmetry. An approximate (isotropic) treatment of cell e.s.d.'s is used for estimating e.s.d.'s involving l.s. planes.
Refinement. Refinement of *F*^2^ against ALL reflections. The weighted *R*-factor *wR* and goodness of fit *S* are based on *F*^2^, conventional *R*-factors *R* are based on *F*, with *F* set to zero for negative *F*^2^. The threshold expression of *F*^2^ > σ(*F*^2^) is used only for calculating *R*-factors(gt) *etc*. and is not relevant to the choice of reflections for refinement. *R*-factors based on *F*^2^ are statistically about twice as large as those based on *F*, and *R*- factors based on ALL data will be even larger.

### Fractional atomic coordinates and isotropic or equivalent isotropic displacement parameters (Å^2^)

**Table:**

	*x*	*y*	*z*	*U*_iso_*/*U*_eq_	
Cu	0.34936 (2)	0.003603 (15)	0.032956 (16)	0.03079 (8)	
O1	0.51708 (12)	0.08258 (8)	−0.01885 (10)	0.0349 (3)	
O2	0.65975 (14)	0.18355 (10)	−0.03382 (12)	0.0509 (4)	
C1	0.54722 (19)	0.15958 (13)	−0.02762 (13)	0.0335 (4)	
C2	0.4344 (2)	0.22176 (13)	−0.03118 (15)	0.0390 (5)	
H2A	0.3904	0.2116	0.0211	0.047*	
H2B	0.4716	0.2775	−0.0244	0.047*	
C3	0.33151 (19)	0.21773 (12)	−0.12042 (15)	0.0371 (4)	
C4	0.3645 (3)	0.18987 (17)	−0.20253 (17)	0.0583 (7)	
H4A	0.4507	0.1714	−0.2029	0.070*	
C5	0.2707 (3)	0.1891 (2)	−0.2844 (2)	0.0775 (9)	
H5A	0.2943	0.1704	−0.3393	0.093*	
C6	0.1426 (3)	0.2159 (2)	−0.2846 (2)	0.0747 (9)	
H6A	0.0796	0.2149	−0.3394	0.090*	
C7	0.1079 (2)	0.24395 (17)	−0.2039 (2)	0.0633 (8)	
H7A	0.0213	0.2620	−0.2039	0.076*	
C8	0.2023 (2)	0.24547 (14)	−0.12211 (17)	0.0467 (5)	
H8A	0.1786	0.2654	−0.0678	0.056*	
O3	0.43667 (14)	0.02350 (11)	0.16182 (10)	0.0444 (4)	
O4	0.28773 (16)	−0.06110 (11)	0.20372 (11)	0.0555 (4)	
C9	0.3872 (2)	−0.01680 (15)	0.22212 (14)	0.0411 (5)	
C10	0.4592 (2)	−0.00598 (19)	0.32311 (16)	0.0596 (7)	
H10A	0.5128	−0.0550	0.3420	0.071*	
H10B	0.5188	0.0414	0.3269	0.071*	
C11	0.3641 (2)	0.00710 (15)	0.38924 (15)	0.0456 (5)	
C12	0.3061 (3)	−0.05839 (18)	0.42846 (19)	0.0650 (7)	
H12A	0.3274	−0.1128	0.4152	0.078*	
C13	0.2172 (3)	−0.0439 (2)	0.4869 (2)	0.0742 (8)	
H13A	0.1795	−0.0887	0.5129	0.089*	
C14	0.1839 (3)	0.0353 (2)	0.50716 (18)	0.0636 (7)	
H14A	0.1238	0.0447	0.5467	0.076*	
C15	0.2394 (3)	0.10011 (19)	0.46888 (19)	0.0631 (7)	
H15A	0.2170	0.1543	0.4821	0.076*	
C16	0.3284 (3)	0.08655 (17)	0.41081 (18)	0.0563 (6)	
H16A	0.3655	0.1319	0.3854	0.068*	
N1	0.23495 (15)	−0.01459 (10)	−0.09298 (11)	0.0314 (3)	
N2	0.19920 (15)	0.08350 (10)	0.04221 (11)	0.0325 (3)	
C17	0.2643 (2)	−0.06336 (14)	−0.16069 (14)	0.0391 (5)	
H17A	0.3461	−0.0906	−0.1511	0.047*	
C18	0.1775 (2)	−0.07459 (15)	−0.24405 (15)	0.0466 (5)	
H18A	0.2003	−0.1090	−0.2898	0.056*	
C19	0.0565 (2)	−0.03411 (16)	−0.25860 (16)	0.0495 (6)	
H19A	−0.0037	−0.0413	−0.3141	0.059*	
C20	0.0255 (2)	0.01729 (14)	−0.18992 (15)	0.0427 (5)	
H20A	−0.0553	0.0456	−0.1988	0.051*	
C21	0.11684 (18)	0.02584 (12)	−0.10760 (13)	0.0312 (4)	
C22	0.09470 (17)	0.07952 (12)	−0.02961 (13)	0.0306 (4)	
C23	−0.02197 (19)	0.12337 (13)	−0.02906 (15)	0.0383 (5)	
H23A	−0.0940	0.1183	−0.0781	0.046*	
C24	−0.0295 (2)	0.17477 (14)	0.04555 (16)	0.0440 (5)	
H24A	−0.1070	0.2047	0.0473	0.053*	
C25	0.0786 (2)	0.18136 (14)	0.11732 (16)	0.0452 (5)	
H25A	0.0763	0.2170	0.1671	0.054*	
C26	0.1906 (2)	0.13390 (14)	0.11387 (15)	0.0405 (5)	
H26A	0.2626	0.1371	0.1631	0.049*	
O5	0.2460 (2)	−0.23227 (13)	0.20341 (15)	0.0773 (6)	
H51	0.2485	−0.1839	0.2272	0.116*	
H52	0.2711	−0.2215	0.1520	0.116*	

### Atomic displacement parameters (Å^2^)

**Table:**

	*U*^11^	*U*^22^	*U*^33^	*U*^12^	*U*^13^	*U*^23^
Cu	0.02265 (12)	0.03791 (15)	0.03088 (13)	0.00331 (9)	0.00258 (8)	−0.00214 (10)
O1	0.0285 (6)	0.0323 (7)	0.0433 (8)	0.0024 (5)	0.0051 (6)	0.0023 (6)
O2	0.0342 (8)	0.0455 (9)	0.0739 (11)	−0.0039 (7)	0.0121 (7)	0.0069 (8)
C1	0.0320 (10)	0.0356 (11)	0.0312 (10)	0.0014 (8)	0.0019 (8)	−0.0005 (8)
C2	0.0400 (11)	0.0334 (11)	0.0426 (11)	0.0032 (9)	0.0055 (9)	−0.0029 (9)
C3	0.0351 (10)	0.0321 (10)	0.0428 (11)	0.0030 (8)	0.0039 (8)	0.0058 (9)
C4	0.0551 (14)	0.0698 (18)	0.0468 (14)	0.0191 (13)	0.0007 (11)	−0.0008 (13)
C5	0.090 (2)	0.090 (2)	0.0448 (15)	0.0176 (18)	−0.0081 (14)	−0.0041 (15)
C6	0.0710 (19)	0.075 (2)	0.0636 (19)	0.0014 (16)	−0.0254 (15)	0.0121 (15)
C7	0.0376 (12)	0.0606 (17)	0.086 (2)	0.0029 (11)	−0.0042 (13)	0.0222 (15)
C8	0.0377 (11)	0.0428 (12)	0.0599 (14)	0.0023 (9)	0.0099 (10)	0.0093 (11)
O3	0.0309 (7)	0.0674 (10)	0.0337 (8)	0.0018 (7)	0.0031 (6)	−0.0036 (7)
O4	0.0500 (9)	0.0661 (11)	0.0491 (10)	−0.0099 (8)	0.0062 (7)	−0.0053 (8)
C9	0.0315 (10)	0.0574 (14)	0.0335 (11)	0.0116 (10)	0.0037 (8)	−0.0074 (10)
C10	0.0410 (12)	0.100 (2)	0.0355 (12)	0.0106 (13)	0.0012 (9)	−0.0053 (13)
C11	0.0457 (12)	0.0607 (15)	0.0286 (10)	0.0041 (11)	0.0024 (9)	0.0002 (10)
C12	0.090 (2)	0.0494 (15)	0.0579 (16)	0.0079 (14)	0.0187 (15)	0.0018 (13)
C13	0.096 (2)	0.071 (2)	0.0629 (18)	−0.0154 (18)	0.0345 (16)	0.0074 (16)
C14	0.0631 (16)	0.085 (2)	0.0467 (15)	−0.0023 (15)	0.0211 (12)	−0.0068 (15)
C15	0.0716 (18)	0.0637 (17)	0.0549 (16)	0.0097 (14)	0.0144 (13)	−0.0113 (13)
C16	0.0660 (16)	0.0532 (15)	0.0509 (14)	−0.0050 (12)	0.0140 (12)	0.0018 (12)
N1	0.0259 (7)	0.0354 (9)	0.0325 (8)	0.0004 (6)	0.0044 (6)	−0.0008 (7)
N2	0.0259 (7)	0.0372 (9)	0.0340 (8)	0.0012 (7)	0.0046 (6)	−0.0020 (7)
C17	0.0321 (10)	0.0462 (12)	0.0395 (11)	0.0043 (9)	0.0080 (8)	−0.0070 (9)
C18	0.0477 (12)	0.0546 (14)	0.0372 (11)	0.0004 (11)	0.0071 (9)	−0.0115 (10)
C19	0.0462 (13)	0.0617 (15)	0.0358 (12)	0.0001 (11)	−0.0044 (9)	−0.0055 (11)
C20	0.0333 (10)	0.0501 (13)	0.0413 (12)	0.0052 (9)	−0.0018 (9)	0.0010 (10)
C21	0.0260 (9)	0.0332 (10)	0.0341 (10)	−0.0011 (7)	0.0048 (7)	0.0023 (8)
C22	0.0256 (8)	0.0330 (10)	0.0330 (10)	−0.0009 (8)	0.0052 (7)	0.0034 (8)
C23	0.0282 (9)	0.0416 (11)	0.0440 (11)	0.0037 (8)	0.0040 (8)	0.0025 (9)
C24	0.0323 (10)	0.0449 (12)	0.0565 (14)	0.0080 (9)	0.0125 (9)	−0.0031 (10)
C25	0.0416 (11)	0.0467 (13)	0.0492 (13)	0.0034 (10)	0.0135 (10)	−0.0125 (10)
C26	0.0339 (10)	0.0462 (12)	0.0405 (11)	0.0011 (9)	0.0046 (8)	−0.0085 (10)
O5	0.0807 (14)	0.0718 (13)	0.0769 (14)	−0.0005 (11)	0.0082 (11)	0.0222 (11)

### Geometric parameters (Å, °)

**Table:**

Cu—O3	1.9558 (15)	C12—H12A	0.9300
Cu—O1^i^	1.9808 (14)	C13—C14	1.363 (4)
Cu—N1	2.0107 (17)	C13—H13A	0.9300
Cu—N2	2.0234 (16)	C14—C15	1.356 (4)
Cu—O1	2.3668 (14)	C14—H14A	0.9300
O1—C1	1.286 (2)	C15—C16	1.372 (4)
O1—Cu^i^	1.9808 (14)	C15—H15A	0.9300
O2—C1	1.231 (2)	C16—H16A	0.9300
C1—C2	1.518 (3)	N1—C17	1.340 (3)
C2—C3	1.517 (3)	N1—C21	1.351 (2)
C2—H2A	0.9700	N2—C26	1.340 (3)
C2—H2B	0.9700	N2—C22	1.352 (2)
C3—C4	1.381 (3)	C17—C18	1.380 (3)
C3—C8	1.389 (3)	C17—H17A	0.9300
C4—C5	1.388 (4)	C18—C19	1.377 (3)
C4—H4A	0.9300	C18—H18A	0.9300
C5—C6	1.377 (4)	C19—C20	1.382 (3)
C5—H5A	0.9300	C19—H19A	0.9300
C6—C7	1.371 (4)	C20—C21	1.386 (3)
C6—H6A	0.9300	C20—H20A	0.9300
C7—C8	1.390 (3)	C21—C22	1.481 (3)
C7—H7A	0.9300	C22—C23	1.385 (3)
C8—H8A	0.9300	C23—C24	1.382 (3)
O3—C9	1.272 (3)	C23—H23A	0.9300
O4—C9	1.228 (3)	C24—C25	1.379 (3)
C9—C10	1.533 (3)	C24—H24A	0.9300
C10—C11	1.508 (3)	C25—C26	1.383 (3)
C10—H10A	0.9700	C25—H25A	0.9300
C10—H10B	0.9700	C26—H26A	0.9300
C11—C16	1.380 (3)	O5—H51	0.8494
C11—C12	1.383 (4)	O5—H52	0.8560
C12—C13	1.379 (4)		
			
O3—Cu—O1^i^	90.90 (6)	C13—C12—C11	120.8 (3)
O3—Cu—N1	171.79 (6)	C13—C12—H12A	119.6
O1^i^—Cu—N1	95.55 (6)	C11—C12—H12A	119.6
O3—Cu—N2	92.68 (7)	C14—C13—C12	120.8 (3)
O1^i^—Cu—N2	174.44 (6)	C14—C13—H13A	119.6
N1—Cu—N2	80.52 (7)	C12—C13—H13A	119.6
O3—Cu—O1	89.67 (6)	C15—C14—C13	119.1 (3)
O1^i^—Cu—O1	77.72 (6)	C15—C14—H14A	120.5
N1—Cu—O1	96.65 (6)	C13—C14—H14A	120.5
N2—Cu—O1	106.54 (6)	C14—C15—C16	120.7 (3)
C1—O1—Cu^i^	118.61 (12)	C14—C15—H15A	119.7
C1—O1—Cu	138.41 (12)	C16—C15—H15A	119.7
Cu^i^—O1—Cu	102.28 (6)	C15—C16—C11	121.5 (3)
O2—C1—O1	123.49 (18)	C15—C16—H16A	119.3
O2—C1—C2	120.32 (19)	C11—C16—H16A	119.3
O1—C1—C2	116.18 (17)	C17—N1—C21	118.68 (17)
C3—C2—C1	113.63 (17)	C17—N1—Cu	126.20 (13)
C3—C2—H2A	108.8	C21—N1—Cu	115.11 (13)
C1—C2—H2A	108.8	C26—N2—C22	118.62 (16)
C3—C2—H2B	108.8	C26—N2—Cu	126.61 (13)
C1—C2—H2B	108.8	C22—N2—Cu	114.59 (13)
H2A—C2—H2B	107.7	N1—C17—C18	122.32 (19)
C4—C3—C8	118.3 (2)	N1—C17—H17A	118.8
C4—C3—C2	121.36 (19)	C18—C17—H17A	118.8
C8—C3—C2	120.3 (2)	C19—C18—C17	119.0 (2)
C3—C4—C5	120.9 (2)	C19—C18—H18A	120.5
C3—C4—H4A	119.6	C17—C18—H18A	120.5
C5—C4—H4A	119.6	C18—C19—C20	119.4 (2)
C6—C5—C4	120.1 (3)	C18—C19—H19A	120.3
C6—C5—H5A	120.0	C20—C19—H19A	120.3
C4—C5—H5A	120.0	C19—C20—C21	118.9 (2)
C7—C6—C5	120.0 (2)	C19—C20—H20A	120.6
C7—C6—H6A	120.0	C21—C20—H20A	120.6
C5—C6—H6A	120.0	N1—C21—C20	121.75 (19)
C6—C7—C8	119.9 (2)	N1—C21—C22	114.69 (16)
C6—C7—H7A	120.1	C20—C21—C22	123.56 (18)
C8—C7—H7A	120.1	N2—C22—C23	121.76 (18)
C3—C8—C7	120.9 (2)	N2—C22—C21	114.49 (16)
C3—C8—H8A	119.5	C23—C22—C21	123.75 (17)
C7—C8—H8A	119.5	C24—C23—C22	118.88 (19)
C9—O3—Cu	114.65 (14)	C24—C23—H23A	120.6
O4—C9—O3	124.2 (2)	C22—C23—H23A	120.6
O4—C9—C10	120.3 (2)	C25—C24—C23	119.50 (19)
O3—C9—C10	115.5 (2)	C25—C24—H24A	120.3
C11—C10—C9	112.61 (19)	C23—C24—H24A	120.3
C11—C10—H10A	109.1	C24—C25—C26	118.7 (2)
C9—C10—H10A	109.1	C24—C25—H25A	120.6
C11—C10—H10B	109.1	C26—C25—H25A	120.6
C9—C10—H10B	109.1	N2—C26—C25	122.46 (19)
H10A—C10—H10B	107.8	N2—C26—H26A	118.8
C16—C11—C12	117.1 (2)	C25—C26—H26A	118.8
C16—C11—C10	120.3 (2)	H51—O5—H52	100.6
C12—C11—C10	122.5 (2)		

Symmetry codes: (i) −*x*+1, −*y*, −*z*.

### Hydrogen-bond geometry (Å, °)

**Table:**

*D*—H···*A*	*D*—H	H···*A*	*D*···*A*	*D*—H···*A*
O5—H51···O4	0.85	2.05	2.781 (3)	143
O5—H52···O2^i^	0.86	2.08	2.931 (3)	174
C20—H20A···O4^ii^	0.93	2.38	3.245 (3)	156
C24—H24A···O2^iii^	0.93	2.48	3.172 (3)	131
C25—H25A···O5^iv^	0.93	2.50	3.201 (3)	132

Symmetry codes:  (i) −*x*+1, −*y*, −*z*; (ii) −*x*, −*y*, −*z*; (iii) *x*−1, *y*, *z*; (iv) −*x*+1/2, *y*+1/2, −*z*+1/2.
